# Estimating the whole-body effective dose and health risks as well as introducing a new easy method for eye lens dosimetry in interventional cardiology procedures

**DOI:** 10.1016/j.mex.2020.101097

**Published:** 2020-10-10

**Authors:** Alireza Hatami, Mahmoud Bagheri, Farzaneh Falahati, Amin Banaei, Razzagh Abedi-Firouzjah, Hamed Zamani, Mohammad Kiapour, Farideh Momeni

**Affiliations:** a Department of Medical Physics, Faculty of Medicine, Shahid Sadoughi University of Medical Sciences, Yazd, Iran; b Research Center for Molecular and Cellular Imaging, Tehran University of Medical Sciences, Tehran, Iran; c Department of Medical Physics and Biomedical Engineering, Faculty of Medicine, Tehran University of Medical Sciences, Sina Campus, Tehran, Iran; d Department of Medical Physics, Faculty of Medical Sciences, Tarbiat Modares University, Tehran, Iran; e Cellular and Molecular Research Center, Yasuj University of Medical Sciences, Yasuj, Iran; f Department of Medical Physics Radiobiology and Radiation Protection, School of Medicine, Babol University of Medical Sciences, Babol, Iran; g Medical Physics and Medical Engineering Department, School of Medicine, Shiraz University of Medical Sciences, Shiraz, Iran

**Keywords:** Dose estimation, Eye lens dosimetry, Whole-body dose, Interventional Cardiology, Cancer risk

## Abstract

This study aimed to introduce a new method for eye lens thermo-luminescent dosimetry and also estimate the dose associated with induced cancer risk due to the ionizing radiation exposure received by physicians and other staff cooperating in interventional cardiology (IC) procedures. The measurements were performed with six TLDs (thermoluminescent dosimeters): four TLDs for eye lens dosimetry (2 positioned on respiratory/surgical mask under the eye region as the new method; and 2 near the outside border of the eye as the common method) and two TLDs for whole-body dosimetry. Whole-body doses were used to calculate the cancer risks induced by IC procedures. The results of the new proposed method for eye lens dosimetry were similar to common TLD positioning (mean differences <5%) and mask displacement had no significant effect on eye dose measurement in our new method. Our proposed method for eye lens dosimetry is simpler and more comfortable compared to the common method and it can be used as an alternative method without using TLD holders to monitor lens dose for IC workers wearing masks during the procedure. The estimated excess cancer incidence risk induced by IC procedures was 29.58 ± 5.71 and 46.68 ± 7.77 (per 100000 individuals) for men and women, respectively.

## Graphical abstract

**Figure:**
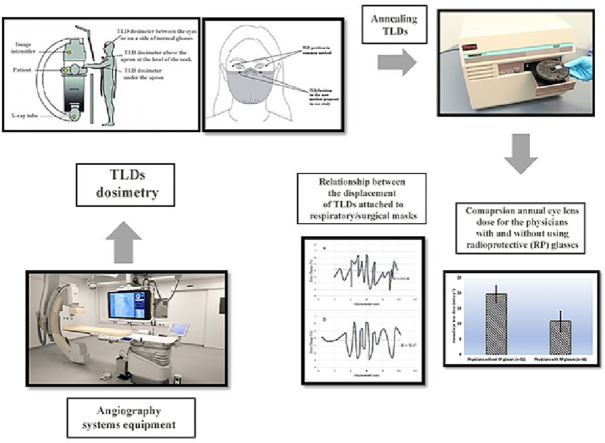

Specifications Table

**Table:** 

Subject Area:	Medicine and Dentistry
More specific subject area:	Evaluating the annual eye lens and whole-body dose, introducing a new easier method for eye lens TLD dosimetry, and also estimating excess cancer risks induced by annual irradiation on IC workers*.*
Protocol name:	Eye lens TLD dosimetry using attached TLDs on respiratory/surgical masks in IC workers
Reagents/tools:	TLD-100 made of LiF: Mg, Cu, P (Thermo, Ohio, USA) and Harshaw TLD reader 5500 (Thermo Scientific, USA).
Experimental design:	Eight general hospitals were chosen for measurements. Eye lens and whole-body dose exposure were monitored in 80 physicians and 80 staff with introducing a new eye lens dosimetry method (new TLD positioning). TLD readouts were converted to absorbed dose using NCRP (National Commission of Radiation Protection) Report No. 122 [1]. Excess cancer risks induced by annual irradiation were estimated regarding the previous studies [2,3].
Trial registration:	“IR.SSU.MEDICINE.REC.1395.297”
Ethics:	In this study, we did not perform any invasive techniques on the patients/staff. The research was approved by Shahid Sadoughi University of Medical Sciences (Yazd, Iran).
Method name:	Lens dosimetry using mask
Value of the Protocol:	• The proposed method for eye lens dosimetry is easier and more comfortable compared to the common method. • This protocol can be used as an alternative method without using TLD holders to monitor lens dose for IC workers wearing masks during the procedure. • Future research and experiments can use this method for studying the health effects of radiation on staff cooperating in radiological procedures.

## Description of protocol

### Background information

Estimating the personal effective dose (E) and eye lens dose for staff working in IC (interventional cardiology) procedures remains necessary and important, although there have been studies previously conducted on this subject [4], [5], [6], [7]. In addition, it must be performed in different regions to ensure radiation safety and health risks regarding ICRP (international commission of radiation protection) recommendations [8,9].

Using body dosimeters are relatively easy and comfortable. However, due to the scatter radiations from body, direct estimation of eye dose is not possible using whole body dosimeters. Therefore, eye dosimeters must be attached to the skin near the eye or positioned by specific holders which make their application hard and limited. Thus, a new method for eye lens dosimetry was introduced employing, respiratory/surgical masks as TLD (thermoluminescent dosimeter) holders.

## Experimental design and introducing a new method for eye lens TLD dosimetry

160 individuals participated in the current study, namely 80 physicians and 80 participants from other staff including nurses, radiologist technologists, anesthesia technologists, and other health professionals at 8 general hospitals (Tehran, Iran). All procedures were performed in cardiac catheterization laboratories, and all measurements were collected from July to October 2019. During the measurements, the IC workers wore the same protective lead aprons as they usually wear.

A new TLD positioning method was introduced for eye lens dosimetry. Respiratory/surgical masks were used as TLD holders in this method which makes the eye dosimetry easier and more useable.

In the common method which has been described in more details by Principi et al. [10], four TLDs for eye lens dosimetry are positioned near the left and right outer side of eyes using TLD holders which fixed with a taping around the head.

**Fig. 1:**
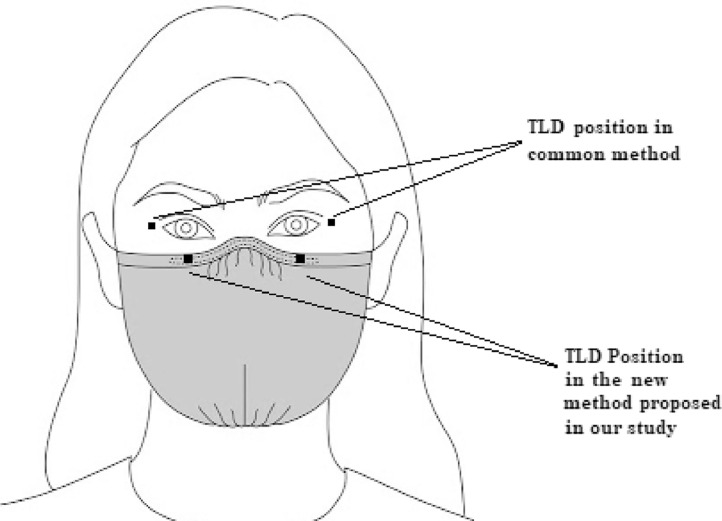
Positions of TLDs attached on the top border of respiratory/surgical mask below the eye region as our new protocol for eye lens dose measurement in comparison with TLD positioned near eyes as common method.

In the new proposed method, since almost all the staff in the assessed centers used mask during their work, our new protocol for eye lens dosimetry consists of attaching two TLDs to the top border of the respiratory/surgical mask below the eye region, as illustrated in Fig. 1. The mask displacement from the initial position was recorded for all participants at the end of IC procedure (by marking the positions on participants’ skin) to evaluate the effect of TLD displacement in eye lens measurement. Furthermore, eye lens dose values obtained from the proposed protocol were compared with the values resulting from the common method. It must be mentioned that our new TLD dosimetry method was just assessed on participants who did not wear radioprotective (RP) glasses.

For both common and new eye lens dosimetry methods, one TLD was positioned on top of the apron and another one, under the apron on the chest regions according to NCRP report 122 [1] for whole-body dosimetry.

## TLD reading and dose measurement

Doses were recorded using TLDs (TLD-100 chips, Harshaw Chemical). The dosimeters consist of LiF:Mg,Cu,P thermo-luminescent detectors with a diameter of 4.5 mm, a thickness of 0.8 mm, and a density of 2.65 g.cm^−3^. The dosimeters were calibrated at Iran Secondary Standard Dosimetry Laboratory (ISSDL) to convert the readouts to Hp (10) and Hp (3) for whole-body and eye lens dosimetry, respectively. Before the measurements, the dosimeters were annealed in a TLD annealing furnace (1 h at 400°C and 2 h at 100°C) and prior to the readout, the dosimeters were pre-heated at 100°C for 20 min.

Generally, the personal dose equivalent, Hp (d), is an operational quantity for individual monitoring. According to the ICRU Publication 103 [9], the personal dose equivalent is defined as “the dose equivalent in soft tissue as defined in ICRU 51 [11] at an appropriate depth, d, below a specified point on the human body”. The Hp (d) obtained from TLD reading after the calibration procedure represents personal dose equivalent from external exposure in the depth of d. The common method for obtaining the Hp (d), is based on air kerma measurements with a standard dosimeter like TLD positioned in air and using conversion coefficients to Hp (d) when the dosimeter is placed on the surface of the ISO slab, pillar or rod standard phantoms. Hp (10) and Hp (3) values were used for estimating the personal whole-body and eye lens absorbed doses, respectively [1,^9^,12]. We used the reported conversion coefficients from air kerma to Hp (3) for eye lens dose assessment as calculated in a cylinder calibration phantom which is close to the mass and shape of a human head for reference photon radiations [13], [14], [15].

The NCRP-122 report offers a calculation of the absorbed dose by giving weight to the dosimeter readouts [1]. In this report, the Rosenstein and Webster algorithm [16] was used as the conversion factor for E/Hp (10) for the photon energies used in radiological examinations in various radiation fields. In addition, this report recommends weighting factors for Hp (10) readout values obtained from two attached dosimeters, to calculate the absorbed dose. The method used in this research has been outlined in equation 1.

$$Equation\;1:\;E\left(estimate\right)=0.025H_{p}{\left(10\right)}_{over}+0.5H_{p}{\left(10\right)}_{under}$$

Where H_P_ (10)_over_ and H_P_ (10)_under_ are the TLD readouts obtained from the dosimeters on the chest (front) and under the apron, respectively. E (estimate) is the estimated effective dose in the above equation.

Hp (3) is the main quantity for monitoring the eye lens dose as recommended by international commission on radiological protection [8]. Thus, initially, the doses were measured in terms of Hp (3) using TLDs. The eye lens dose was calculated by averaging the number of Hp (3) from two TLDs readout values for each participant.

## BEIR VII phase 2 model for estimating cancer risks

The BEIR VII phase 2 (Biological Effects of Ionizing Radiation Version2, phase 2) reports the committee's preferred estimates of the lifetime attributable risk of incidence and mortality for all solid cancers and for leukemia for low dose radiations (Tables 1–13 of BEIR VII phase 2 report) [2]. We used the values of the coefficients in this report and the estimated annual average absorbed dose to calculate the risks for men and women.

## Number of IC procedures and using of RP glasses

The number of IC procedures that any of the physicians/staff had cooperated during the last year was obtained from each hospital. The annual absorbed dose for each participant was estimated by IC absorbed dose values from one procedure and the total number of procedures in one year.

In the current study, 48 physicians used RP glasses and 32 ones did not use the glasses during the IC producers. The annual eye lens dose for the two groups was calculated for comparison.

## Statistical analysis

The annual eye lens dose for two groups (with and without RP glasses) were compared using independent t-test statistical analysis (SPSS 16, IBM, USA). Furthermore, in participants without eye RP glasses, a comparison was carried out between the eye lens dose values of the common method and our new proposed method, using a paired t-test. The resulting P-values lower than 0.05 waere considered as statistically significant differences.

## Extraction of the data

Tables 1 and 2 represent the estimated mean ± SD eye lens dose (both for our new proposed method and common method), and whole-body annual effective dose, respectively, for physicians and staff in the eight hospitals.

**Table 1:** The mean ± SD annual values of IC procedures, estimated eye lens dose, and whole-body effective dose for the physicians (interventional radiologists and cardiologists) in various hospitals (10 physicians in each hospital).

Hospital No.	Annual number of IC procedures	Annual absorbed eye lens dose received from IC procedure in common method (mGy)		Annual absorbed eye lens dose received from IC procedure in our new method (mGy)		Annual effective whole-body dose received from IC procedure (mSv)
		Left eye	Right eye	Left eye	Right eye	
Hospital 1	205.2 ± 32.6	18.83 ± 4.15	20.41 ± 5.54	17.33 ± 3.83	19.19 ± 4.32	4.83 ± 0.97
Hospital 2	160.3 ± 20.8	14.08 ± 1.80	12.47 ± 1.91	16.41 ± 2.86	15.78 ± 2.53	3.94 ± 0.45
Hospital 3	154.1 ± 25.3	12.51 ± 1.96	13.11 ± 2.56	11.73 ± 2.38	13.00 ± 1.98	4.73 ± 0.71
Hospital 4	226.1 ± 18.6	12.03 ± 2.31	10.12 ± 2.85	13.22 ± 3.52	12.47 ± 3.14	4.18 ± 0.73
Hospital 5	150.5 ± 40.0	8.99 ± 2.12	10.31 ± 2.54	9.56 ± 2.39	10.33 ± 3.29	3.47 ± 0.70
Hospital 6	192.7 ± 21.2	8.73 ± 1.97	7.17 ± 1.73	7.23 ± 2.24	7.49 ± 2.48	3.64 ± 0.63
Hospital 7	232.1 ± 24.8	12.97 ± 3.27	12.06 ± 4.12	11.75 ± 2.85	10.88 ± 3.13	4.56 ± 0.76
Hospital 8	170.4 ± 33.0	8.86 ± 3.67	9.25 ± 3.08	9.18 ± 3.44	10.18 ± 3.67	3.53 ± 0.67
Overall (Mean ± SD)	186.4 ± 25.9	12.12 ± 2.66	11.86 ± 3.04	12.05 ± 2.94	12.41 ± 3.07	4.11 ± 0.70

**Table 2:** The mean ± SD annual values of IC procedures, estimated eye lens absorbed dose, and whole-body effective dose for the other staff (not physicians) in various hospitals (10 staff in each hospital).

Hospital No.	Annual number of IC procedures	Annual absorbed eye lens dose received from IC procedure in common method (mGy)		Annual absorbed eye lens dose received from IC procedure in our new method (mGy)		Annual effective whole-body dose received from IC procedure (mSv)
		Left eye	Right eye	Left eye	Right eye	
Hospital 1	260.9 ± 33.5	3.71 ± 0.50	4.04 ± 0.87	3.44 ± 0.66	3.84 ± 0.66	2.83 ± 0.52
Hospital 2	202.7 ± 26.6	3.16 ± 0.29	2.89 ± 0.38	3.31 ± 0.37	3.01 ± 0.53	2.51 ± 0.48
Hospital 3	191.1 ± 38.2	3.02 ± 0.59	3.09 ± 0.55	3.11 ± 0.41	3.23 ± 0.60	2.71 ± 0.49
Hospital 4	250.4 ± 23.2	3.42 ± 0.60	3.12 ± 0.61	3.37 ± 0.56	3.19 ± 0.74	2.36 ± 0.42
Hospital 5	170.9 ± 15.6	2.89 ± 0.79	3.05 ± 0.67	2.75 ± 0.82	2.90 ± 0.77	2.42 ± 0.81
Hospital 6	216.2 ± 30.3	2.62 ± 0.71	2.45 ± 0.85	2.72 ± 0.66	2.38 ± 0.62	2.27 ± 0.61
Hospital 7	231.6 ± 28.0	3.36 ± 0.63	3.83 ± 0.74	3.29 ± 0.70	3.57 ± 0.88	2.86 ± 0.63
Hospital 8	247.6 ± 19.3	2.60 ± 0.91	2.81 ± 0.75	2.68 ± 0.88	2.94 ± 0.78	2.40 ± 0.61
Overall (Mean ± SD)	221.4 ± 26.9	3.10 ± 0.63	3.16 ± 0.68	3.08 ± 0.63	3.13 ± 0.70	2.55 ± 0.57

The cancer risks (all solid tumors+leukemia) induced by annual whole-body doses, from IC procedures for physicians and other staff have been presented in Table 3.

**Table 3:** The average of cancer incidence and mortality risks induced by annual whole-body effective dose from IC procedures for physicians and other staff

	Induced cancer risk (per 100,000)	Death risk due to induced cancers (per 100,000)
Physicians (Male:55, Female:25)	Male: 35.79 ± 6.34 Female: 58.61 ± 7.74	Male: 18.73 ± 3.36 Female: 28.14 ± 4.62
Other staff (Male:49, Female:31)	Male: 22.95 ± 5.13 Female: 34.93 ± 7.81	Male: 12.24 ± 2.74 Female: 16.50 ± 3.76
Overall (Mean ± SD)	Male: 29.58 ± 5.71 Female: 46.68 ± 7.77	Male: 15.65 ± 3.21 Female: 22.12 ± 4.38

**Fig. 2:**
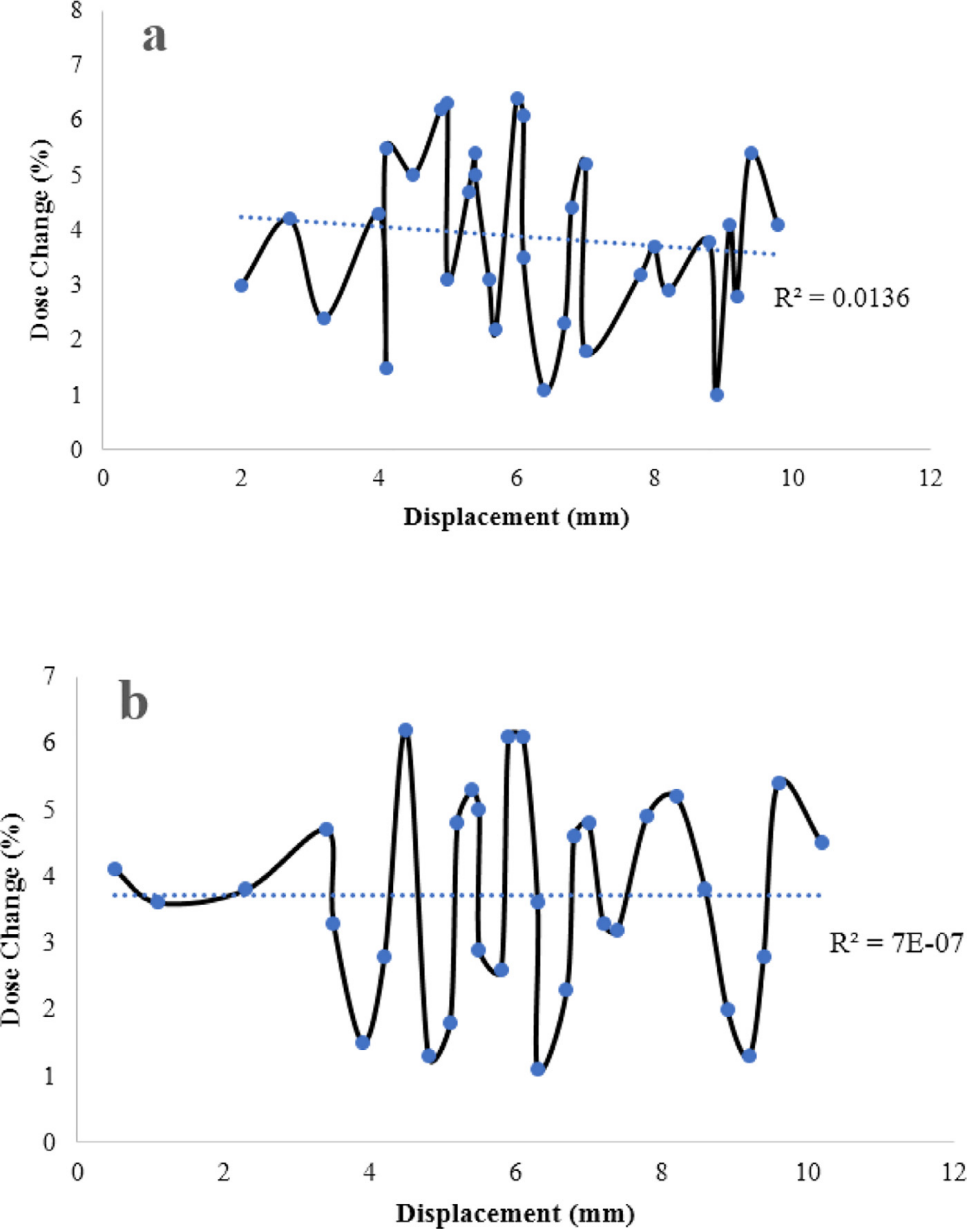
The relationship between the displacement of TLDs attached to respiratory/surgical masks during IC procedure and percentage of dose change compared to theTLD measurement positioned near the eye, for left (a) and right (b) eyes. Dash lines and R^2^ values represent the linear fitting to the data.

The statistical analysis for TLD eye lens dosimetry comparing the common and our new method showed no significant differences using paired t-test (*p*-value = 0.03). Fig. 2 represents the relationship between the different displacement of TLDs on surgical masks and the percentage of dose change. The eye dose values did not differ significantly from common TLD measurements (mean difference < 4%, maximum difference < 7%) at different displacement measurements. Therefore, it can be concluded that mask movement in IC procedures had not affected the eye lens dose measurement, significantly. Table 4 illustrated the differences between our new proposed method and the common method for eye lens dosimetry.

**Table 4:** The qualitative differences between the new proposed method and common method for eye lens thermo-luminecense dosimetry

Location of the TLDs	New proposed method	Common method
	Attached on the respiratory/surgical masks under the eyes	Left or right side of the eye positioned on TLD holders
Needing holder or wrap	No	Yes
Safe for user	Yes	Yes
Comfortability	Comfortable	Not comfortable
Cost	Lower cost	Higher cost

**Fig. 3:**
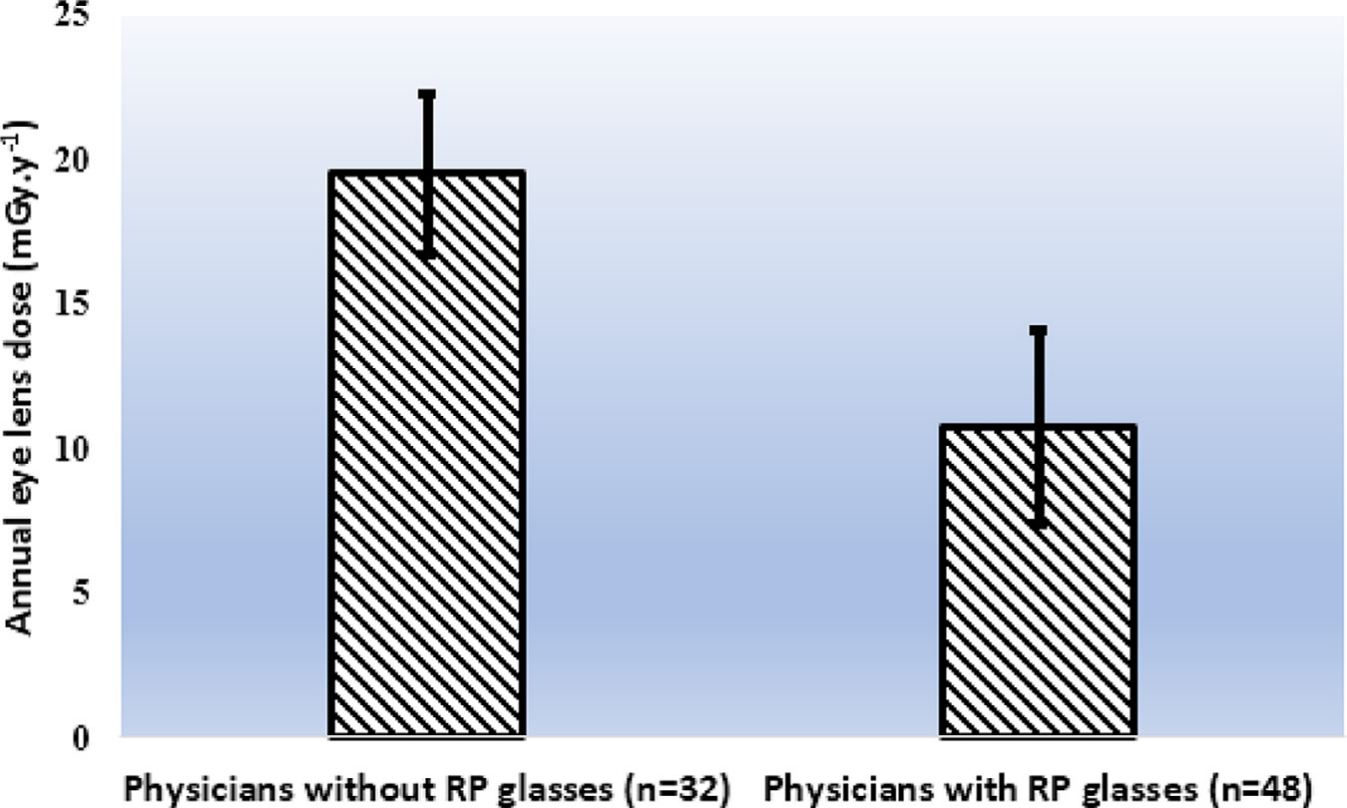
Mean ± SD (as error bars) values of the annual eye lens dose (mGy.y^−1^) for the physicians using radioprotective (RP) glasses in comparison with the physicians without using RP glasses in IC procedures.

The annual absorbed eye lens dose (mGy) from IC procedures between the physicians who used the RP glasses (n = 48) and those who did not RP glasses (n = 32) has been shown in Fig. 3. The statistical analysis using independent t-test showed that physicians using RP glasses had lower annual absorbed eye lens dose (*p* < 0.02).

## Conclusion

In the current study, a new method was introduced for eye lens dosimetry which is simpler and more comfortable compared to the common protocol. Our results showed that the dosimetry readings with this method were consistent with the previous methods, therefore, it can be used as an alternative method for IC workers wearing respiratory/surgical masks. Furthermore, the new method is not sensitive to mask displacement.

Another finding of the current study revealed that the annual estimated effective dose for staff working in IC procedures is under the values (20 mSv) proposed by ICRP reports; in addition, the induced cancer risks are relatively small, however, the eye lens dose could be significantly decreased using RP glasses.

## Declaration of Competing Interest

The authors declare that they have no known competing financial interests or personal relationships that could have appeared to influence the work reported in this paper.
